# Literacy Acquisition Trajectories in Bilingual Language Minority Children and Monolingual Peers with Similar or Different SES: A Three-Year Longitudinal Study

**DOI:** 10.3390/brainsci12050563

**Published:** 2022-04-27

**Authors:** Paola Bonifacci, Ida Carmen Ferrara, Jessica Pedrinazzi, Francesco Terracina, Paola Palladino

**Affiliations:** 1 Department of Psychology, University of Bologna, 40126 Bologna, Italy; 2 Department of Brain and Behavioral Sciences, University of Pavia, 27100 Pavia, Italy; ida.ferrara@hotmail.it (I.C.F.); pedrinazzi.jessica@gmail.com (J.P.); francesco.terracina@libero.it (F.T.); 3 Learning Science Hub, Department of Humanities, University of Foggia, 71122 Foggia, Italy; paola.palladino@unifg.it

**Keywords:** bilingualism, reading, spelling, reading comprehension, listening comprehension, language minority bilingual children

## Abstract

Bilingualism and socio-economic status (SES) differentially affect linguistic and cognitive development. However, less evidence has been collected regarding their impact on literacy trajectories. The present longitudinal study evaluated the literacy development of language minority bilingual children (LMBC) and monolingual peers with different SES. A group of LMBC with low-SES (*n* = 18) and monolingual peers with low (*n* = 18) or high (*n* = 14) SES were followed from 2nd to 5th grade through a set of tasks assessing decoding (words, nonwords, passage), reading, and listening comprehension, and spelling skills. The results showed that all groups achieved better performances over time in all measures, except listening comprehension. However, low-SES LMBC underperformed in spelling tasks compared to the monolingual groups. In reading comprehension, there was a time*group interaction that showed how low-SES LMBC reached similar performances of low-SES monolinguals in fifth grade, but both groups underperformed compared to the high SES monolingual group. The discussion is focused on the need for research and educational settings to consider the differential impact of bilingualism and SES. Bilingualism seems to be associated with a longer time in developing adequate spelling skills, whereas SES was the primary underpinning of the reading comprehension gap over time.

## 1. Introduction

The socio-political conditions of the most recent years have increased migrations: millions of migrants live outside their country of birth. All these people are faced with the task of acquiring a language that is different from the one spoken at home, and all children attending school also develop literacy skills in a second language. These children might be defined as bilingual children within a functional conception of bilingualism that refers to the use and need of two or more languages in everyday life [1]. However, most children of migrant families are exposed to the second language at school after being exposed to the first language at home, and often, families have a low socio-economic status (SES).

In Italy, it is estimated that students from migrant backgrounds represent 10.3% of the school population [2]. The median income for migrant families is about 56% of that of families with both Italian parents, and the composite index of being at-risk for poverty and social exclusion is 56.8% for families with both non-Italian parents, compared to 23.4% of families with Italian parents [3]. The Italian welfare system allows free access to healthcare and education and, for Italian students, SES has been reported to have a minor predictive role in academic achievements compared to other European countries [4]. However, parents’ level of instruction and profession may mediate access to different living conditions, as well as different exposures to verbal exchanges and involvement in dialogues and reading experiences. Previous evidence found that, in the Italian context, SES was related to early literacy skills in monolingual populations, and it was mediated by home literacy and numeracy activities [5]. Therefore, the differential influence of sequential bilingualism and SES on literacy development needs to be better investigated.

Bilingualism and socio-economic status (SES) differentially affect linguistic and cognitive development [6,7]. A study conducted in the Italian context [8] found that, after controlling for quantity of bilingual exposure, variation in SES was predictive of vocabulary, grammar, and working memory skills. On the other hand, the variation in bilingual exposure predicted vocabulary and narrative comprehension more effectively than the variation in SES. However, this study did not include literacy skills. The majority of studies that compared language minority bilingual children (LMBC) reading development to the monolingual peers control group, were cross-sectional. Even though they have shed light on literacy skills at a single point in time, they have failed to provide insight into the process of literacy development over time and how it compares to that of monolingual peers. Furthermore, the role of SES in the comparison of bilingual and monolingual literacy performances was rarely considered in longitudinal studies. Finally, language orthographic transparency might impact literacy acquisition, both in monolingual and bilingual populations. In transparent orthographies, such as Italian, reading accuracy at the end of first grade was around 95%, but it was less than 75% in the deeper orthographies [9]; a similar trend was found for spelling accuracy at the end of second grade [10]. Acquiring a transparent orthography might also be a facilitating factor for bilingual children. However, the bilingual’s gap in literacy skills might be more consistent for transparent vs. opaque languages, since LMBC children might take longer to reach full reading and spelling accuracy, also in light of their reduced vocabulary size.

The present study evaluated the developmental trajectories of language minority bilingual children and monolingual peers with different SES in word and nonword reading, passage reading, and oral comprehension, spelling (orthographic) skills in fifth grade, following a previous study in which they were tested in second grade [11].

### 1.1. Literacy Skills in Bilinguals

Since the seminal contribution of [12], the main evidence suggests that, after two years of schooling in English as a second language, language minority bilingual children reach monolingual-like performance in word and nonword reading. Similar results have been found in bilingual children exposed to transparent languages, although on some occasions, differences in word reading have been found for words of increasing orthographic complexity [13], where bilingual children underperformed compared to their monolingual peers. In studies on the Italian language, a similar trend was found for low frequency, or irregular words [14]; late bilinguals, characterized by a smaller L2 vocabulary size, were less accurate than early bilinguals and monolinguals in assigning non-dominant stress. Studies conducted on either transparent [15] or opaque [16,17] orthographies found that bilinguals may fall behind their monolingual peers in word and text reading fluency, potentially because they tend to adopt the sublexical/phonological route instead of the lexical one, given the well-documented slower lexical access [18,19,20], and/or smaller receptive vocabularies [21]. A study that compared low-SES bilinguals and low- and high-SES monolingual children in the Italian context did not report differences across groups in word, nonword, and passage reading [11].

Several studies have reported that bilinguals appear to lag behind monolingual peers in reading comprehension tasks (for a meta-analysis: [22]). However, some studies showed that bilinguals could reach reading comprehension skills comparable to those observed in monolingual groups in opaque orthographies [23,24,25] and in transparent languages [15,26]. Age of first exposure might be a relevant factor; for example, [15] found that early bilinguals did not differ in reading comprehension scores compared to monolingual peers, but there was a significant difference between late bilinguals and monolinguals. With reference to the Simple View of Reading [27,28], which states that reading comprehension can be considered the product of decoding and oral language comprehension skills, most evidence suggests that bilinguals’ weaknesses in reading comprehension rest mainly on verbal language skills, including vocabulary size, rather than on decoding [22,29,30].

Considering spelling, a meta-analysis [31] pointed out that bilinguals outperformed monolinguals in real-word spelling tasks, while monolinguals seem to have better performances in nonword spelling tasks. Authors explained these results suggesting that bilinguals may be learning to spell from print, therefore learning accurate spelling for real words, not pseudo-words. However, studies on children exposed to L2 transparent languages depict a different pattern. A study [13] on bilingual children exposed to Dutch in the first two grades of primary school reported that they underperformed compared to monolinguals in word spelling. Another study [32] found discrepancies in spelling tasks between bilinguals and monolinguals until the end of primary school, while the differences were no longer significant from 6th grade. Polish and Turkish students exposed to German [33] were more likely to be classified as poor spellers in 3rd–4th grade than in higher grades. Studies involving LMBC learning Italian as a second language [34] found that fourth-fifth graders underperformed in words, nonwords and passage dictation writing tasks compared to their monolingual peers and [11], reported that second graders language-minority bilinguals underperformed compared to monolinguals in a dictation task, even when controlling for socio-economic status.

In summary, results from cross-sectional studies, mainly conducted on children in primary school, suggest that LMBC might reach adequate word and nonword decoding skills within the first two years of schooling. However, they might underperform compared to monolinguals in complex/low-frequency word decoding, reading text fluency, reading comprehension, and spelling, at least for words and texts.

### 1.2. Longitudinal Research on Literacy Development in Bilinguals

A study [35] examined longitudinally the differences between MLBC and monolingual peers on component measures of reading from kindergarten to fourth grade exposed to English as L2. They showed that although some differences in reading measures may be found in pre-schoolers, in fourth-grade differences are negligible, and the two groups appear to show very comparable skills even for reading comprehension. In another study [36] conducted with 261 Latino kindergarten children, decoding scores remained in the average range compared to the normative English-speaking sample throughout the data collection period. However, as regards reading comprehension, it began to fall behind the normative sample starting in the third grade. In a longitudinal study [37] from preschool to 11th grade, focused on Spanish-English bilingual children, it was found that word reading skills were on par with English monolinguals, but reading comprehension was lower across all time points. Following longitudinally bilingual and monolingual children from grade 4th to grade 6th [38] found that L2 children outperformed L1 readers in word and pseudoword decoding but had significantly lower reading comprehension scores than L1 readers across grades. In a study [39] called the Early Childhood Longitudinal Study, Kindergarten (ECLS-K) project involving bilingual and monolingual children over the elementary years, bilinguals underperformed at first, third, and fifth grades in reading comprehension measures but not in decoding measures, compared to monolinguals. Reading comprehension was also investigated by a cohort-sequential design study focused on a sample of Spanish-speaking minority language bilingual students within two academic years [40]. Measures of vocabulary breadth, vocabulary depth, morphological awareness, syntactic skill, and reading comprehension were obtained twice a year for two consecutive academic years. In a synthesis of a complex pattern, results showed that minority language bilingual children fall below their monolingual peers in all the literacy indices. However, once all language predictors were controlled within the model, the differences in literacy skills between groups disappeared. This specific result was difficult to be explained, and its generalization may shed light on the relationship investigated. Authors indicated that “…Future studies should include bilingual students from multiple language groups (e.g., [17]) and disentangle how language skills may be related to reading comprehension for students from a variety of different language backgrounds.” In another longitudinal study, Reference [41] studied a large sample of Spanish-speaking minority language bilinguals to examine their literacy development from third to fifth grade. Bilinguals’ performances were compared to the entire population of California students using State standardized test data, which included word reading fluency and vocabulary, literary response, writing, and grammar. Results demonstrated that bilingual minority language children scored below average in many literacy indices compared to the overall State population. As grade increases bilinguals made slight gains to narrow the gap with monolinguals. The bilinguals’ highest performance was obtained between fourth and fifth grade but plateaued after that. These results suggest that literacy learning is a more difficult task for bilingual minority language children than for monolinguals; bilinguals obtained performance level at the fourth/fifth grade about 15% lower than monolinguals. However, according to California cut-off, the authors indicated that Spanish bilinguals examined in this study were for 92% of the sample below the poverty level. Therefore, the comparison was likely to be largely biased by SES level.

Regarding spelling, Reference [42] found that bilingual children underperformed monolingual peers in simple spelling in kindergarten but outperformed monolinguals in word and nonword spelling when they reached 2nd grade.

In summary, most longitudinal evidence suggests that language minority bilingual children reach adequate decoding skills over the first years of schooling. However, contrasting results emerge regarding the development of reading comprehension, possibly because more intervening factors, such as SES, might influence the developmental trajectory. It is to note that relatively little longitudinal evidence has been collected on spelling and listening comprehension. Furthermore, only a few studies included SES in group matching.

### 1.3. The Present Study

The study’s main aim was to trace the developmental trajectories of three groups of children, LMBC, and monolinguals with low and high SES, from second to fifth grade in literacy acquisitions: reading, spelling, reading, and listening comprehension. The longitudinal study may clarify whether the learning gaps observed in second grade between groups (bilingual compared to monolingual with low SES, but also bilinguals and monolinguals with low SES compared to monolinguals with high SES) may be at least partially overcome in three more years of school, third, fourth and fifth grade. Furthermore, comparable performances also deserve attention to check whether the overlapping is maintained or if any change is observable with school attendance and learning increases. According to literature, and in agreement with previous results [11], different predictions can be sketched on the domains of literacy development tested in the present study:

(1) For decoding skills, we expected groups to exhibit comparable performances in reading fluency. However, literature found that reading speed and accuracy for words and nonwords can reach monolingual-like levels after two years of immersive scholastic exposure [12]. Therefore, the development of decoding skills through primary school years is not expected to be affected by bilingualism and SES; however, differences might emerge in passage reading [15,16,17], which involves vocabulary knowledge and lexical access.
(2) For reading comprehension skills, we expected that bilingualism and SES might negatively impact the developmental trajectory. Previous studies indicated that BMLC perform below monolinguals in reading comprehension, and contrasting results have been found for listening comprehension [11,15]. Therefore, SES’s role may also be relevant in affecting literacy acquisition.
(3) Regarding spelling skills, previous studies conducted on LMBC exposed to a transparent language found significant differences compared to monolingual peers [11,26,32,33,34], but no effect of SES was found. We, therefore, expect that LMBC might underperform compared to monolingual children in spelling tasks, independently of SES.

The comparison between groups of low SES bilinguals and low and high SES monolinguals may disentangle the effect of bilingualism and the role of SES. If LMBC will perform below monolinguals with low SES, it is possible to refer the learning delay to bilingualism. However, if LMBC perform comparably to monolinguals with low SES but lower than monolinguals with high SES, SES mainly be responsible of the gap.

## 2. Materials and Methods

### 2.1. Participants

The sample included 50 children (54% female; *M* age T2 = 10.31 years, DS = 0.30) who were enrolled in the study in the second half of 2nd grade and who were then followed longitudinally with a second assessment in the second half of 5th grade of primary school. The whole sample, according to the criteria defined below, was divided into three groups: (1) bilingual language minority children with low SES (*n* = 18); (2) monolingual children matched for gender, age, and SES level (*n* = 18); (3) a group of monolingual children matched for gender and age but with higher SES (*n* = 14).

Results from the first measurement time point are also reported in [11]. Sample attrition was 13.7% in fifth grade, the main reason is moving to a different school. Children were selected from three classes in a suburb area of a northern Italian region, which has the highest absolute number of foreign students in Italy, according to [2]. Italian was the unique language of instruction for all children, except for 2 h of an English course in the school setting. None of the participants had received a clinical diagnosis of neurodevelopmental disorders.

Monolingual children were exposed both at home and at school to Italian. Bilinguals had been exposed to an L1 other than Italian (L2) within the family context from birth and to the Italian language through at least two years of immersive scholastic exposure; none of them had been schooled in their L1. The L1 languages included Arabic (38.9%), Albanian (16.6%), Chinese (5.6%), Russian (11.1%), Pidgin-French (11.1%), Spanish (5.6%), Pidgin-English (11.1%). and. Most of them were born in Italy (94.4%), and one child arrived in Italy within the first two years of age. All the bilingual children attended infant school in Italy, and 50% also attended nursery school.

Children’s language proficiency in L1 was assessed through parents’ rating through the QUBil questionnaire (see method section for a detailed description). Parents responded on a 5-points scale (0 = absence of skill; 5 = fully developed skill), and all children resulted in having good L1 language proficiency, with a mean value of 3.9 (SD = 0.83) for language comprehension and 4.18 (SD = 0.75) for expressive oral language skills. For Italian language proficiency, a similar rating scale has been proposed to teachers, and also in this case, scores were in the high range: 4.27 (SD = 1.27) for comprehension; 4.45 (SD = 0.82) for oral expression. The profile of bilingual competence that emerged from parents’ and teachers’ reports confirms that the bilingual sample had effective knowledge of the L1 and adequate skills in L2 oral language, which allowed them to perform the tasks proposed in the present study.

All bilingual children showed a low to medium SES, as measured by the Hollingshead Four Factor Index of Social Status [43]. Monolingual children were divided into two groups based on SES level: a group (*n* = 18) with low to medium SES and a group (*n* = 14) with high SES. In Table 1 mean values and statistics about group differences in SES score and age are reported.

### 2.2. Materials

Linguistic history: The Questionnaire on the bilingual pupils’ linguistic history (QUBil) is included in the “BaBIL” battery [44]. It’s an interview for parents to collect information on children’s linguistic history. There are questions about the following subjects: each language learning starting time in the child’s personal history, languages known by the child, the amount of each language use in and out of the family context, level of proficiency perceived by parents and teachers in L1 and L2. The interview served to ascertain children’s linguistic history and respect inclusion and exclusion criteria. Data from the interview were selected, and the following were considered in the dataset: age of exposure, year of arrival in Italy, parents and teachers perceived proficiency in L1 and L2.

Socio-economic level (SES): The Hollingshead Four Factor Index of Social Status [43] has been adopted in the current study. The indexes of educational level (EL) and occupation (O) were used. Educational level is scored between 1 and 7, and occupation with scores from 1 to 9. The formula EL *x* 3 + O *x* 5 was adopted to obtain SES scores for fathers and mothers; the compound SES score for children resulted from the mean of the two values. Final scores between 0 and 39 were classified as low-medium and those above 40 as medium-high.

Decoding and reading comprehension tasks: The ALCE battery (Assessment Lettura e Comprensione in età Evolutiva [Assessment of Reading and Comprehension skills in Developmental Age] [45] is a standardized battery for the assessment of reading and comprehension skills in children from first to fifth grade of primary school. Words (*n* = 60), nonwords (*n* = 30), and a passage reading task are included, along with listening comprehension and a reading comprehension task. Reading speed was measured in syllables per second (s/s), whereas a percentage of errors was calculated for the total number of words the children read. For reading comprehension, children are asked to read a passage and respond to 10 open questions presented orally and scored on a 0–2 points scale (maximum score: 20). For listening comprehension, the experimenter read a text followed by 10 questions assessing comprehension; these were scored as for the reading comprehension task. Raw scores were converted into T-scores according to the test manual. A full description of the task and reliability indexes can be found in [46].

Spelling test: Spelling has been tested through the dictation of a short passage entitled “La ricetta delle pere allo sciroppo” [The recipe for pears in syrup], which is part of a standardized battery “Prove ZERO” [Zero Battery] [47] for the early identification of learning disabilities. The text is composed of 54 words (including articles and prepositions), which represented spelling rules typical of Italian orthography, such as double letter within words (e.g., zucchero [sugar]), complex bi-and trigrams (eg., sciroppo [syrup]), apostrophes and accents. The test allows verifying the acquisition of the spelling and the stabilization of grapheme-phoneme correspondences. The test score is obtained by counting the total number of words written correctly. Raw scores were converted into z-scores according to test manual.

Some extreme values in z-scores were lowered to a value of—5sd for all groups. This allows keeping track of the severity level in performance without affecting scores distribution.

### 2.3. Procedure

Parents signed informed consent for participating in the study. Trained psychologists administered the decoding and comprehension tasks individually; the writing task was administered collectively to the class. The individual testing session lasted approximately 25 min. The collective testing session lasted about 15 min. Breaks were allowed if the child showed signs of fatigue. The same procedure was adopted for both waves of assessment. The study was approved by the Ethics Committee of Psychology, University of Pavia.

## 3. Results

A set of Repeated Measures ANOVAs with Group as between factor variable were run to evaluate developmental trajectories on each separate index of literacy skills considered. Table 2 reports mean values for each task in the two waves and related statistics from the analyses. All variables showed skewness values < ±2 [48]. The analyses were performed using IBM SPSS Statistics for Mac, Version 26.0 (Armonk, NY: IBM Corp). All analyses were conducted on T-scores or z-scores (for spelling), computed with reference to normative values appropriate for age, taken from tests’ manuals. This allows considering developmental trajectory about what is expected for each age range considered and not absolute values. Gender differences in the whole sample were evaluated through t-tests on each variable included in the study, and no significant difference emerged (all *p*s > 0.05).

### 3.1. Decoding Skills

**Figure 1:**
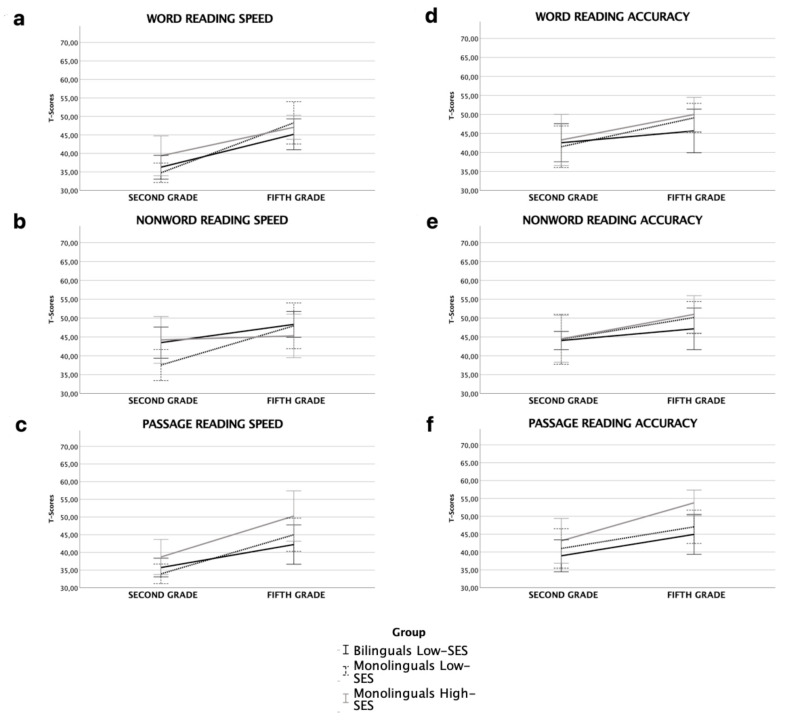
Line bars representing decoding skills (T-scores) in word reading speed (**a**), nonword reading speed (**b**), passage reading speed (**c**), word reading accuracy (**d**), nonword reading accuracy (**e**) and passage reading accuracy (**f**) in second and fifth grade for the three groups (error bars: 95% CI).

Trajectories in decoding skills are reported in Figure 1a–f. Considering word reading there was a main effect of Time [F_(2,47)_ = 83,53, *p* <.001, n^2^_p_ = 0.64], but no effect of group [F_(2,47)_ = 0.49, *p* = 0.62, n^2^_p_ = 0.02], nor for the interaction Time*Group [F_(2,47)_ = 2,64, *p* = 0.8, n^2^_p_ = 0.10]. The same trend was observed for word reading accuracy, with a main effect of Time [F_(2,47)_ = 11.77, *p* = 0.001, n^2^_p_ = 0.20], but no effect of Group [F_(2,47)_ = 0.38, *p* = 0.68, n^2^_p_ = 0.02] nor for the interaction Time*Group [F_(2,47)_ = 0.71, *p* = 0.19, n^2^_p_ = 0.03].

As regards nonword reading speed there was an effect of Time [F_(2,47)_ = 26.27, *p* < 0.001, n^2^_p_ = 0.36], no effect of group [F_(2,47)_ = 0.6, *p* = 0.55, n^2^_p_ = 0.03], but a significant interaction Time*Group emerged [F_(2,47)_ = 6.43, *p* < 0.01, n^2^_p_ = 0.21]. Low-SES monolinguals underperformed compared to the other two groups at T1 but the differences were not still significant at T2. There was no effect of Group [F_(2,47)_ = 0.32, *p* = 0.73, n^2^_p_ = 0.01] or interaction Time*group [F_(2,47)_ = 0.43, *p* = 0.66, n^2^_p_ = 0.02] in nonword reading accuracy, although it remained a significant effect of Time [F_(2,47)_ = 10.36, *p* < 0.01, n^2^_p_ = 0.18]. Finally, with reference to text reading speed there was an effect of Time [F_(2,47)_ = 63.3, *p* < 0.001, n^2^_p_ = 0.18], but no effect of Group [F_(2,47)_ = 2.27, *p* = 0.11, n^2^_p_ = 0.08] nor interaction Time*Group [F_(2,47)_ = 1.81, *p* = 0.17, n^2^_p_ = 0.07]. The same pattern was observed for text reading accuracy: Group [F_(2,47)_ = 2.23, *p* = 0.12, n^2^_p_ = 0.09]; Time [F_(2,47)_ = 34.14, *p* < 0.001, n^2^_p_ = 0.42], Time*Group interaction [F_(2,47)_ = 1.29, *p* = 0.28, n^2^_p_ = 0.05].

### 3.2. Listening and Reading Comprehension

**Figure 2:**
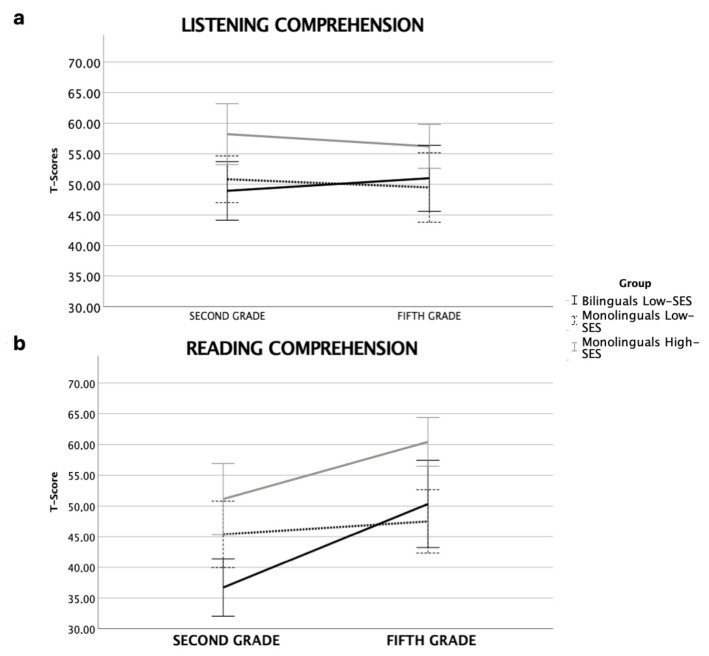
Line bars representing listening (**a**) and reading comprehension (**b**) performance (T-scores) in second and fifth grade for the three groups (error bars: 95% CI).

Trajectories in listening and reading comprehension skills are reported in Figure 2a,b. In listening comprehension (Figure 2a), there was no effect of Time [F_(2,47)_ = 0.11, *p* = 0.75, n^2^_p_ = 0.002], no significant interaction time *x* Group [F_(2,47)_ = 0.93, *p* = 0.40, n^2^_p_ = 0.04] and no effect of group [F_(2,47)_ = 0.6, *p* = 0.55, n^2^_p_ = 0.03]. Conversely, in the reading comprehension task (Figure 2b), main effects of Time [F_(2,47)_ = 30.17, *p* < 0.001, n^2^_p_ = 0.39], Group [F_(2,47)_ = 7.3, *p* < 0.01, n^2^_p_ = 0.24] and of the interaction Time*Group [F_(2,47)_ = 5.31, *p* < 0.01, n^2^_p_ = 0.18] were found. The group effect showed that low-SES bilinguals and monolinguals did not differ, but both underperformed compared to high-SES monolingual children. However, the time* group interaction showed that low-SES bilinguals had poorer reading comprehension scores at T1 with respect to both low- and high-SES monolinguals groups, who did not differ from each other. At T2, the two low-SES groups did not differ, but both underperformed compared to high-SES children.

### 3.3. Spelling

**Figure 3:**
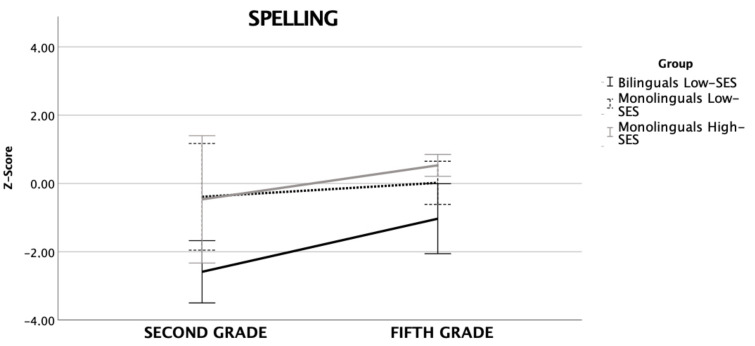
Line bars representing spelling performance (z-scores) in second and fifth grade for the three groups (error bars: 95% CI).

Trajectories in spelling skills are reported in Figure 3. Regarding spelling there was a main effect of time [F_(2,47)_ = 8.35, *p* < 0.01, n^2^_p_ = 0.15] and a significant effect of group [F_(2,47)_ = 4.92, *p* < 0.01, n^2^_p_ = 0.17] but no interaction time *x* group [F_(2,47)_ = 1.03, *p* = 0.37, n^2^_p_ = 0.04]. Post-hoc tests (Bonferroni) showed that low-SES bilinguals underperformed compared to low-SES monolinguals (*p* < 0.05) and high-SES monolinguals (*p* < 0.05), which did not differ from one another.

## 4. Discussion

The present longitudinal study investigated literacy development trajectories from second to fifth grade of language minority bilingual children compared to two groups of monolingual peers, one with similar low-SES and one with high-SES. The study aimed to examine and compare the three groups’ learning trajectories to better understand the role of home language (bilingualism) and SES level on literacy acquisition in primary school. Results showed that language minority bilingual children learning progress in reading speed and accuracy in words, nonwords, and passage reading were comparable to that of their monolingual peers, independently from SES. They were able to learn decoding skills as their peers from second grade and consistently gained in speed and accuracy scores to fifth grade as their peers. It has to be noted that all groups had relatively low scores in second grade, with mean T-scores between 38 and 42, and for all groups, there was a main effect of Time, with final scores fully in the norms from 46 to 50. Therefore, the study demonstrated that reading fluency acquisition in a transparent language during primary school overlaps for language minority bilingual children and their monolingual peers. Results on word and nonword reading align with previous studies that documented the absence of a main gap in decoding skills for bilinguals with at least two years of regular school exposure [11,12,14,15,26]. However, these results partially differ from some studies that documented significant differences between monolinguals and bilinguals in passage reading [15,16,17]. Since the present sample was relatively homogenous in the first years of primary school, with High-SES monolinguals performing slightly below the national average, all children might have developed more complex decoding skills at the same pace, reducing the likelihood of developing group differences in decoding skill.

Similarly, SES did not result in differentiating monolingual children in decoding skills during primary school, according to previous studies [11,49]. However, it has to be underlined that there was a significant interaction time*group in nonword reading speed, where low-SES monolinguals started from a lower level of performance and showed a considerable increase compared to the other group. For this group, we might speculate that the Low-SES condition might have impacted a poorer home literacy environment, which is connected to early linguistic and literacy skills [5].

Differently from what was reported in the first wave of assessment [11], in the present sample, which was subject to attrition in sample recruitment, there were no differences amongst the three groups in listening comprehension, neither at Time 1 nor at Time 2. Listening comprehension was expected to be mainly influenced by SES, given the well-known association between SES and linguistic development [50]. However, from a qualitative point of view, there was a difference in fifth grade of around 10 points in terms of T-scores between the two low-SES groups and the high-SES group.

In more complex literacy skills such as reading comprehension, bilingualism and SES seem relevant, although with different patterns. Both low-SES groups underperformed in reading comprehension compared to the high-SES group, both in second and fifth grades. However, low-SES bilinguals had worse performance in second grade than monolingual low-SES peers but showed a better improvement over time in reading comprehension than low-SES monolinguals. If bilingualism appeared the most impacting factor on reading comprehension in second grade, three years later, in fifth grade, bilingualism appeared no longer significant. SES seemed to be the condition that may primarily impact reading comprehension performance at this school level. As previously discussed, most studies in the literature report a disadvantage in reading comprehension in bilinguals, even considering longitudinal trajectories [38,39,41]. Although the results from the present study might confirm this pattern, they add an important insight into the need to consider SES in group matching since the “bilingual gap” in reading comprehension might be better explained as an “SES gap”. Recent studies in the Italian context highlighted the relevance of SES in the literacy skills of primary school children, and for bilingual children, it was found that SES impacts precursors of reading comprehension skills such as vocabulary, grammar, and working memory skills [8]. Since SES affects cognitive and control skills, such as working memory and executive functions, fundamental in supporting literacy acquisition [51,52], these skills might also be crucially involved in developing second language literacy skills [53,54,55].

Finally, considering spelling skills, a main effect of bilingualism emerged, since bilinguals underperformed from second to third grade compared to low- and high-SES children. These results confirm previous evidence from studies on bilingual children exposed to a transparent language [11,26,32,33,34]. This result indicates that spelling seems to be affected by home language rather than by SES level. However, it has to be considered that these results partially differ from others conducted on opaque languages [31]. Indeed, children exposed to transparent languages develop their writing skills faster than those exposed to opaque languages [56], and writing in the Italian language has been reported to be strongly influenced by lexical skills [57]. Therefore, monolingual children may achieve good performances, mainly through lexical strategies, in relatively short times. This might heighten the bilingual gap in spelling skills, particularly for a highly transparent orthography such as Italian since bilinguals might rely more on phonological skills than on lexical processing, due to their limited vocabulary in L2 [21].

The relatively small sample size is a limitation of the study, and future longitudinal studies should involve larger samples. Further, and connected to, bilingual populations are highly heterogeneous, and a more fine-grained evaluation of the precise relationship between literacy trajectories and time of exposure, linguistic distance between L1 and L2, and home literacy activities would allow for setting more accurate predictions. In addition, it would be important to include groups of bilingual language minority children with High-SES in further studies. In the present study, SES was measured considering parents’ educational and occupational levels. Further indices might be included in future studies that consider other more sensitive parameters of SES level in populations with migrant backgrounds, with particular reference to the access to resources of the national welfare.

Finally, further studies on wider samples should include different statistical analyses such as regressions and structural equation models to better understand which factors are the main contributors to reading and spelling performance.

## 5. Conclusions

To sum up, results from the present study depict a complex picture of literacy development in relation to SES and bilingualism. None of these factors impacted decoding skills in word, nonword and text, nor do they impact listening comprehension. However, SES rather than bilingualism was the discriminating factor in reading comprehension skills at the end of primary school. On the counterpart, bilingualism, but not SES, was the key factor of spelling disadvantage over the primary school years.

The present results represented an original effort to disentangle the role of SES and bilingualism in affecting literacy skills acquisition during primary school, particularly for considering different subdomains of literacy skills such as decoding, listening and reading comprehension, and spelling. It also allows enriching data on literacy development in bilingual and monolingual populations exposed to a highly transparent language such as Italian.

From a research perspective, the present study results reinforce the need to include SES in bilingual studies accurately and further understand the complex interaction between SES and bilingualism in learning processes. Moreover, these results might have clinical implications, suggesting that poor spelling in bilingualism might not necessarily correspond to a disorder but might be the expression of an extended need for exposure and support. From an educational perspective, this study emphasizes the need to inform teachers about the complex profile of strengths and weaknesses and overcome the “all or nothing” approach that might depict bilingualism as an advantage or a disadvantage. Teachers and educators might benefit from developing clear expectations on the time needed to develop specific literacy skills and the related influence of SES. The present study might suggest offering didactic intervention to support the development of spelling skills in bilinguals and reading comprehension for socially disadvantaged populations.

## Figures and Tables

**Table 1:** Mean and sd scores for demographic variables for the three groups.

Variables	Bilinguals Low-SES		Monolinguals Low-SES		Monolinguals High-SES		Statistics	Post-hoc	n^2^_p_ Effect Size
	Mean	SD	Mean	SD	Mean	SD			
Children’s combined SES	23.1	7.79	28.22	6.31	49.52	5.47	F_(2,49)_ = 66.97, *p* < 0.001	B = MLS < MHS	0.74
Mothers’ SES	22.83	10.29	28	11.99	50.32	6.81	F_(2,49)_ = 31.48, *p* < 0.001	B = MLS < MHS	0.57
Fathers’ SES	23.36	7.76	28.44	9.16	48.71	9.25	F_(2,49)_ = 36.13, *p* < 0.001	B = MLS < MHS	0.61
Age T2	10.25	0.21	10.28	0.36	10.42	0.29	F_(2,49)_ = 1,53, *p* = 0.22		0.06
Gender (% females)	55.55%		61.11%		42.85%		X2(2) = 1.08; *p* = 0.58		

**Table 2:** Mean scores and sd for each literacy task in second and fifth grade for the three groups.

Literacy Measures	Second Grade (T1)						Fifth Grade (T2)					
	Bilinguals Low-SES		Monolinguals Low-SES		Monolinguals High-SES		Bilinguals Low-SES		Monolinguals Low-SES		Monolinguals High-SES	
	Mean	SD	Mean	SD	Mean	SD	Mean	SD	Mean	SD	Mean	SD
Word Reading Speed °	36.28	6.48	34.78	5.29	39.36	9.34	45.17	8.38	48.28	11.5	47.07	5.64
Word Reading Accuracy °	42.56	10.08	41.5	11	43.29	11.66	45.67	11.54	49.11	7.68	50	7.79
Nonword Reading Speed °	43.5	8.33	37.56	8.29	44.21	10.74	48.33	6.87	47.94	12.19	45.29	10
Nonword reading accuracy °	44.06	4.86	44.39	13.31	44.5	10.73	47.17	11.11	50.17	8.54	51	8.58
Text Reading Speed °	35.72	5.3	33.94	5.6	38.71	8.57	42.22	11.16	45	9.46	50.29	12.33
Text Reading Accuracy °	38.94	9.01	41	11.08	43.14	10.85	44.94	11.24	47.06	9.36	53.79	6.18
Text Spelling *	−2.59	1.83	−0.39	3.14	−0.46	3.23	−1.03	2.06	0.02	1.27	0.53	0.56
Listening Comprehension °	48.94	9.65	50.83	7.71	58.21	8.61	51	10.86	49.5	11.42	56.21	6.23
Reading Comprehension °	36.72	9.4	45.39	10.92	51.14	10.03	50.33	14.28	47.5	10.35	60.43	6.85

° T-scores, * Z-scores.

## Data Availability

Data will be made available from authors upon reasonable request.
